# Obstetrics

**Published:** 1904-03

**Authors:** 


Obstetrics. A Text-Book for the Use of Students and Practitioners. By J. Whitridge Williams, Professor of Obstetrics, Johns Hopkins University, etc., Baltimore, Md. With eight colored plates and 630 illustrations and thirty illustrations in the text. Pages, 845. Price, $6.00. New York and London: D. Appleton & Co. 1903.

This volume is one of the year's best products. It is both scientific and practical. It is well illustrated and elaborate, yet nothing seems superfluous. Among the number of standard works on obstetrics, this book has no superior, and few equals. A large field is covered by this volume. because modern views take in a broader scope. The science of obstetrics, as promulgated, has kept pace with other branches of medicine, and all are moving.

